# Unmasking the Threat: Acute Hepatitis E Virus Infection in a Liver Transplant Recipient

**DOI:** 10.1155/crit/5573734

**Published:** 2026-05-20

**Authors:** Mina Awadallah, Avneet Singh, Gillian L. Hale, John Erikson Yap, Yii Chun Khiew, Rebecca Kim, Juan F. Gallegos-Orozco

**Affiliations:** ^1^ Division of Gastroenterology, Hepatology and Nutrition, University of Utah Hospital and Clinics, Salt Lake City, Utah, USA; ^2^ Division of Internal Medicine, Cooper University Hospital, Camden, New Jersey, USA, cooperhealth.org; ^3^ Division of Pathology, University of Utah Hospital and Clinics, Salt Lake City, Utah, USA

## Abstract

Hepatitis E virus (HEV) infection is an increasingly recognized cause of acute and chronic hepatitis in immunosuppressed individuals, including solid organ transplant recipients. Although typically self‐limited in immunocompetent hosts, HEV infection in transplant recipients may lead to persistent viremia, accelerated fibrosis, and graft dysfunction. We report the case of a 47‐year‐old man who underwent deceased donor liver transplantation for decompensated metabolic‐associated steatohepatitis cirrhosis. Five months post‐transplant, he was found to have rising liver enzymes during routine monitoring. Imaging and two liver biopsies showed no evidence of acute cellular rejection. Subsequent evaluation revealed positive HEV IgM and detectable HEV RNA with a viral load of 5.5 million IU/mL. Reduction of immunosuppression and initiation of ribavirin therapy resulted in rapid biochemical improvement and complete viral clearance. HEV RNA remained undetectable at the end of therapy and during extended follow‐up. This case highlights the importance of early consideration of HEV infection in liver transplant recipients with unexplained liver enzyme abnormalities. Prompt diagnosis and guideline‐concordant management can prevent progression to chronic infection and preserve graft function.

## 1. Introduction

Hepatitis E virus (HEV) is a non‐enveloped, single‐stranded RNA virus primarily transmitted via the fecal–oral route. While historically associated with waterborne outbreaks in developing regions, HEV genotypes 3 and 4 are now recognized as important causes of sporadic infection in industrialized countries, typically through zoonotic or foodborne exposure such as undercooked pork, wild game, or processed meat products. HEV infection during pregnancy has been associated with severe disease and high mortality rates, underscoring the virus’s potential clinical significance.

In immunosuppressed individuals, including solid organ transplant recipients, HEV infection may follow a chronic course and lead to accelerated fibrosis, cirrhosis, or graft dysfunction. Immunosuppressive regimens influence viral persistence, with calcineurin inhibitors such as tacrolimus associated with prolonged viremia, while mycophenolate mofetil may exert antiviral effects. International guidelines, including the 2018 European Association for the Study of the Liver (EASL) Clinical Practice Guidelines and the updated 2024 Japanese HEV Guidelines, recommend reduction of immunosuppression as first‐line therapy, followed by ribavirin treatment in cases of persistent viremia.

We present a case of acute HEV infection in a liver transplant recipient that initially mimicked acute cellular rejection, highlighting diagnostic challenges, successful guideline‐based management, and the importance of early HEV testing in the post‐transplant setting.

## 2. Case Presentation

A 47‐year‐old man with decompensated metabolic‐associated steatohepatitis cirrhosis underwent deceased donor liver transplantation at the University of Utah Hospital and Clinics in May 2024. His post‐transplant immunosuppression regimen consisted of tacrolimus 3 mg twice daily and mycophenolate mofetil 720 mg twice daily. He remained abstinent from alcohol and had an initially uncomplicated recovery.

**Figure 1:**
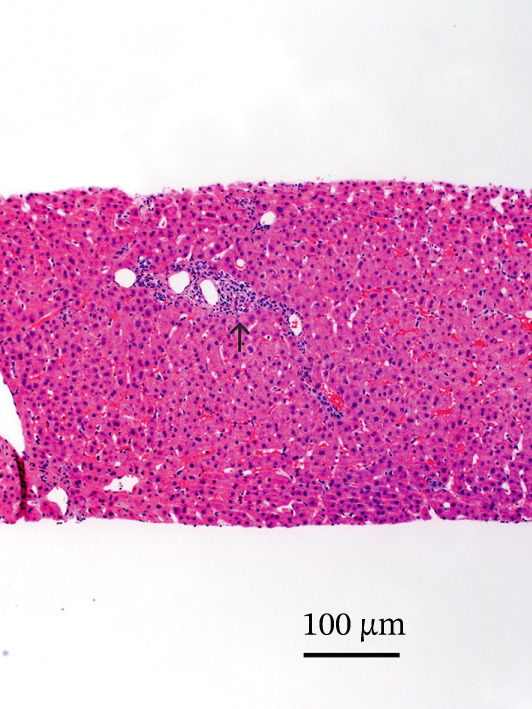
HEV infection in post‐transplant liver biopsy. Hematoxylin and eosin (H&E)‐stained liver core biopsy demonstrating mild portal inflammation composed predominantly of small mature lymphocytes (arrow). Original magnification ×10. Scale bar = 100 *μ*m.

Five months after transplantation, routine outpatient laboratory monitoring revealed rising serum aminotransferases. The patient was asymptomatic at presentation. Magnetic resonance imaging of the abdomen demonstrated patent hepatic vasculature without biliary dilation or obstruction. Given concern for acute cellular rejection, a prednisone taper was initiated while awaiting histopathologic evaluation. Two liver biopsies performed 1 month apart demonstrated mild portal inflammation and ductular reaction but no evidence of acute cellular rejection. Histopathologic findings are illustrated in Figure 1. Serial laboratory trends are summarized in Table 1.

**Table 1:** Laboratory trends after liver transplant. Serial laboratory values demonstrate the progression of liver enzyme abnormalities prior to diagnosis and subsequent biochemical improvement following immunosuppression reduction and ribavirin therapy.

Date	Total bilirubin (mg/dL)	AST (U/L)	ALT (U/L)	Alk Phos (U/L)	Hemoglobin (g/dL)	Creatinine (mg/dL)	Notes
June 2024	0.5	11	29		11.6	1.22	Baseline post‐transplant
August 2024	0.6	92	240		13.4	1.20	Rising transaminases
September 2024	1.1	133	573		14.2	1.47	Pre‐diagnosis peak
October 2024							Ribavirin initiated
October 2024	1.8	186	559		16.3	1.38	Early treatment
October 2024	1.4	105	394		14.0	1.36	Improving
October 2024	1.0	33	89		14.7	1.42	Improving
November 2024	1.0	21	42		14.7	1.32	Near baseline
December 2024	1.3	19	32	69			Continued improvement
December 2024	1.2	24	32	68			Near normalization
December 2024	0.8	20	23	73			**HEV RNA undetectable**

*Note:* The bold entry “HEV RNA undetectable” indicates the time point or clinical status at which hepatitis E virus RNA was no longer detectable in the patient’s serum, reflecting virologic clearance or treatment response.

Despite adjustments to immunosuppression, including tacrolimus dose modifications and subsequent tapering, liver enzymes continued to rise. Comprehensive laboratory evaluation revealed positive anti‐HEV IgM and detectable HEV RNA by polymerase chain reaction, with an initial viral load of 5.5 million IU/mL. Genotype testing was not available, as it is not routinely performed at US centers. Evaluation for other viral etiologies, including hepatitis A and B viruses, cytomegalovirus, Epstein–Barr virus, and human herpesvirus 6, was negative. Dietary history identified recent consumption of commercially purchased jerky, a recognized potential zoonotic source of HEV infection.

Immunosuppression was subsequently reduced, with tacrolimus decreased to 2 mg twice daily and mycophenolate mofetil reduced to 360 mg twice daily. Ribavirin therapy at a dose of 400 mg twice daily was initiated on October 2, 2024. Liver function tests improved steadily following treatment initiation, and serial HEV RNA measurements demonstrated a progressive decline in viral load, becoming undetectable by December 31, 2024.

Ribavirin was discontinued after a 12‐week course. HEV RNA remained undetectable at 12 weeks post‐therapy (March 2025) and during long‐term follow‐up at 6 months (May 2025) and 9 months (August 2025). The patient remained clinically stable, experienced no hospitalizations, and did not develop ribavirin‐related adverse events. A timeline of key clinical events is provided in Table 2.

**Table 2:** Clinical timeline of HEV infection. Timeline outlining key clinical events from liver transplantation through diagnosis, treatment, and long‐term follow‐up of hepatitis E virus infection.

Date	Event
**May 2024**	Deceased donor liver transplantation
June–Sept 2024	Stable recovery; routine outpatient monitoring
**October 2024**	Elevated liver enzymes detected
October 2024	MRI normal; two biopsies show no ACR
**October 2024**	HEV diagnosed; ribavirin started; immunosuppression reduced
Oct–Dec 2024	Biochemical improvement and viral decline
**December 2024**	**HEV RNA undetectable** (end of therapy)
**March 2025**	**12-week sustained virologic response**
May, July, Aug 2025	Continued undetectable HEV RNA
August 2025	Last follow‐up: patient clinically stable

*Note:* The bold entries indicate key virologic milestones during follow‐up. “HEV RNA undetectable (end of therapy)” in December 2024 represents successful viral clearance at completion of antiviral treatment, while “12‐week sustained virologic response” in March 2025 confirms persistent absence of detectable HEV RNA 12 weeks after treatment completion, supporting durable treatment response and viral eradication.

## 3. Discussion

Elevation of aminotransferases in liver transplant recipients frequently raises concern for acute cellular rejection and often leads clinicians to intensify immunosuppressive therapy. However, infectious etiologies should be carefully excluded before escalation of immunosuppression, as viral infections such as HEV may present with similar biochemical abnormalities. HEV infection is increasingly recognized as a cause of unexplained transaminase elevation in solid organ transplant recipients, particularly in developed countries where zoonotic transmission predominates [1, 2].

In immunosuppressed patients, HEV infection may progress to chronic hepatitis within 3 months and may lead to accelerated fibrosis, cirrhosis, and graft dysfunction if untreated [1, 2]. This contrasts with the typically self‐limited course seen in immunocompetent hosts. Solid organ transplant recipients are particularly susceptible due to chronic immunosuppression, which impairs viral clearance and allows persistent viral replication. Calcineurin inhibitors such as tacrolimus have been associated with prolonged viremia, whereas mycophenolate mofetil may exert partial antiviral effects [3].

Management of HEV infection in transplant recipients generally follows a stepwise approach. Current clinical practice guidelines recommend initial reduction of immunosuppression, which alone may result in viral clearance in approximately one‐third of patients [1]. For individuals with persistent viremia, ribavirin therapy for approximately 12 weeks has demonstrated sustained virologic response rates approaching 90%–95% [4]. In our patient, a combination of immunosuppression reduction and ribavirin therapy led to rapid biochemical improvement and sustained viral clearance, consistent with prior reports [2, 4].

Although HEV genotype testing was not available in this case, genotype 3 is the most common cause of zoonotic HEV infection in the United States and other industrialized countries [5]. Processed meat products, including pork and jerky, have been implicated as potential sources of transmission [6]. The patient’s recent consumption of commercially purchased jerky therefore represents a plausible exposure risk.

HEV infection following liver transplantation may be underrecognized, as its clinical presentation can mimic acute rejection or drug‐induced liver injury [1, 2]. In our case, the diagnostic challenge was highlighted by persistently abnormal liver enzymes and two liver biopsies that did not demonstrate rejection. This underscores the importance of considering HEV infection early in the diagnostic evaluation of transplant recipients with unexplained liver enzyme abnormalities.

Routine screening of organ donors for HEV is not currently recommended in the United States. However, targeted testing of transplant recipients with unexplained transaminase elevations may facilitate earlier diagnosis and avoid unnecessary intensification of immunosuppression [2]. Increasing awareness of HEV infection among transplant clinicians is therefore essential to prevent progression to chronic infection and graft injury.

## 4. Conclusion

HEV infection should be considered early in the evaluation of liver transplant recipients presenting with unexplained liver enzyme abnormalities, particularly when histologic findings do not support acute rejection. Early diagnosis, judicious reduction of immunosuppression, and timely ribavirin therapy are highly effective in achieving viral clearance and preventing chronic hepatitis or graft injury. Increased awareness of dietary risk factors and systematic HEV testing may improve outcomes in this vulnerable population.

## Author Contributions

M.A: conceptualization, manuscript writing, editing. A.S, G.L.H: methodology, literature review, editing. J.E.Y, Y.C.K, R.K: Review and editing. J.F.G: supervision, review, editing.

## Funding

No funding was received for this manuscript.

## Ethics Statement

This study was conducted in accordance with institutional ethical standards and the Declaration of Helsinki. Formal institutional review board approval was waived due to the retrospective nature of this case report.

The transplanted organ was obtained in accordance with ethical standards. Written informed consent for organ donation was obtained from the donor’s legal next of kin. The donor was not from an executed prisoner or any vulnerable population, and no coercion was involved in the donation process.

## Consent

Written informed consent was obtained from the patient for publication of this case report and accompanying images and clinical data. All identifying information has been removed to ensure patient confidentiality.

## Conflicts of Interest

The authors declare no conflicts of interest.

## Data Availability

The data that support the findings of this study are available on request from the corresponding author. The data are not publicly available due to privacy or ethical restrictions.
